# An Adaptive 6-DOF Tracking Method by Hybrid Sensing for Ultrasonic Endoscopes

**DOI:** 10.3390/s140609961

**Published:** 2014-06-06

**Authors:** Chengyang Du, Xiaodong Chen, Yi Wang, Junwei Li, Daoyin Yu

**Affiliations:** Key Laboratory of Opto-Electronics Information Technology of Ministry of Education, College of Precision Instrument and Opto-Electronics Engineering, Tianjin University, Tianjin 300072, China; E-Mails: dcy_tju@126.com (C.D.); koala_wy@tju.edu.cn (Y.W.); cd_ljw@126.com (J.L.); dyyu@tju.edu.cn (D.Y.)

**Keywords:** ultrasonic endoscope, orientation, position, hybrid sensing, disturbances

## Abstract

In this paper, a novel hybrid sensing method for tracking an ultrasonic endoscope within the gastrointestinal (GI) track is presented, and the prototype of the tracking system is also developed. We implement 6-DOF localization by sensing integration and information fusion. On the hardware level, a tri-axis gyroscope and accelerometer, and a magnetic angular rate and gravity (MARG) sensor array are attached at the end of endoscopes, and three symmetric cylindrical coils are placed around patients' abdomens. On the algorithm level, an adaptive fast quaternion convergence (AFQC) algorithm is introduced to determine the orientation by fusing inertial/magnetic measurements, in which the effects of magnetic disturbance and acceleration are estimated to gain an adaptive convergence output. A simplified electro-magnetic tracking (SEMT) algorithm for dimensional position is also implemented, which can easily integrate the AFQC's results and magnetic measurements. Subsequently, the average position error is under 0.3 cm by reasonable setting, and the average orientation error is 1° without noise. If magnetic disturbance or acceleration exists, the average orientation error can be controlled to less than 3.5°.

## 1. Introduction

Ultrasonic endoscopes are widely used in examining organs adjacent to the gastrointestinal (GI) track [1–4]. However, with the restrictions of ultrasonic images, identification of anatomical structures is difficult [5]. To assist endoscopic surgeries, navigation systems are desirable. Electro-magnetic tracking (EMT) method has become a common practice in tracking endoscopes, including flexible [5–7] and capsule ones [8–10].

In existing navigation systems for flexible endoscopes, magnetic generators are placed externally, while sensors are attached to the devices. The generators produce specific magnetic fields, measured by the sensors. In a clean environment, magnetic measurements are relatively stable; but when metal objects are near the system, distortions will appear and may significantly affect the measurement results. For instance, Ren [11] has reported that a small metal object can cause 10 degrees of distortions in a NDI (NDI, Waterloo, ON, Canada) EMT device. For a tracking system for ultrasonic endoscopes, the scan head is the major interference source, which will continuously disturb estimates [12]. Equipment like metallic loops, wire guides, and catheters will also influence the outputs. Furthermore, with respect to basic principles, the traditional EMT methods are generally established on approximate models, assuming magnets are dipoles [13,14] or coils are single-term [15]. The processes would undoubtedly introduce measuring errors.

To overcome EMT's sensibility to magnetic distortion, hybrid sensing technologies that fuse EMT with other tracking methods are promising [16]. Commonly, an optical tracking [17,18] or inertial navigation system (INS) [19,20] can be adopted to assist EMT. However, restricted by the line-of-sight conditions [21], integrating EMT and INS becomes the most feasible solution for us, so in our solution, an INS is applied. This subsystem consists of a tri-axis gyroscope and accelerometer, and a magnetic angular rate and gravity (MARG) sensor array. Though the gyroscope can estimate orientations in dynamic motions, its measuring errors grow unbounded because of the bias [22]. At the same time, the gyro-free part—containing the other two sensors—is able to provide drift-free estimation, which can be smoothed by the gyroscope's signals. In the gyro-free part, gravitational acceleration and geomagnetic field are chosen to measure the orientation, where common strategies include TRIAD, QUEST [23], QFA [24] and Gauss-Newton (G-N) [25], and they can also be fused with the outputs of the gyroscope to obtain optimal results through a Kalman filter [26,27]. In all these methods, the sensor data collected by the accelerometer and the magnetometer are regarded as values of gravity and geomagnetic field. Sabatini [28] figured out that there are limitations in such operations, because the outputs of the accelerometer also include accelerated velocities, which are hard to separate, and the outputs of the magnetometer may be distorted by ferromagnetic materials.

To restrain interferences, noises should be considered in estimates. Roetenberg [29] has implemented modeling of the patterns of noises to reduce their influences through a Kalman filter. However, two difficulties restrict application of the method to endoscopic localization. Firstly, the disturbances of sensors are assumed as first-order Markov processes that are driven by white noises. For changeable environments containing multiple noises, the assumptions are not enough to quantify influences of disturbances on orientation estimation. In an operating room, the disturbing sources are various, ranging from metal instruments to electronic equipment, so it is hard to characterize all magnetic disturbances through one uniform model. Secondly, because multiple Markov models are introduced in the filter, the algorithm's complexity is relatively high, which may limit real-time applications. When tracking an endoscope, continuous calculation over a relatively long time is needed, so the speed of the method is also a critical factor. In a word, a more efficient algorithm with anti-jamming capability is needed.

For position determination, integrating acceleration signals [30]—the most common practice in human motion analysis based on INS—is impractical in tracking endoscopes. That is because speed of endoscopes is relative slow when moving *in vivo*, which makes the accelerometer's signals easy to be masked by its noises. Therefore this paper still applies the traditional method in the EMT subsystem—measuring the generators' fields through the magnetometer—for tracking real-time position. Different from typical EMT methods, the results of orientation estimates from the MARG subsystem are employed to reduce dependences on magnetic sensing further and simplify the solving process for position measurements.

The organization of this paper is as follows: Section 2 the gives architecture of our navigation system. In Sections 3 and 4, we present the adaptive fast quaternion convergence (AFQC) and simplified electro-magnetic tracking (SEMT) algorithms. In Section 5, we propose the experimental and evaluation methods. In Section 6, we present and discuss the experimental results related to the localization accuracy, in which anti-interference ability is tested. Finally, conclusions are given.

## 2. System Architecture

**Figure 1.**
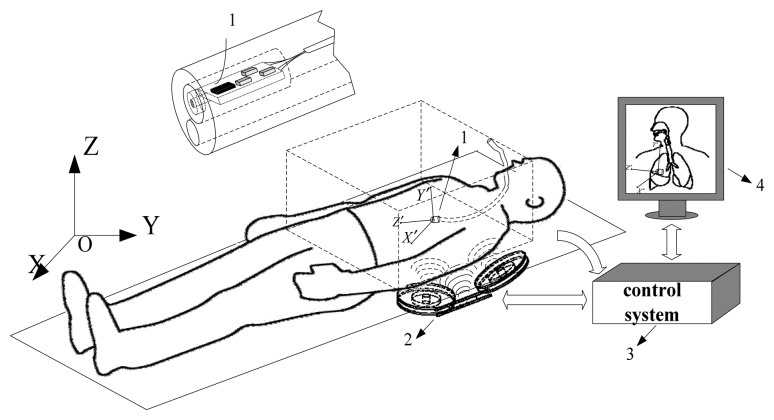
Overview of the ultrasonic endoscope tracking system.

**Figure 2.**
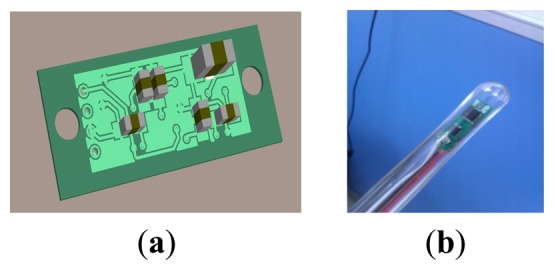
The minimized MARG sensor array. (**a**) The 3D model of the senor array's PCB; (**b**) The photo of the senor array which is sealed in a glass tube.

**Figure 3.**
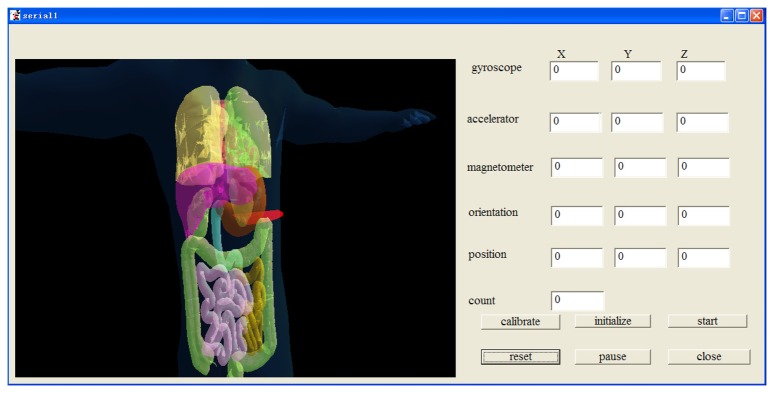
The interface of the ultrasonic endoscope tracking system.

Our overall scheme is shown in Figure 1. The system can be divided into four parts. Part 1 is the MARG sensor, which consists of an InvenSense MPU6050 (InvenSense, San Jose, CA, USA) 16 bit resolution inertial measurement unit (containing a gyroscope and an accelerometer) and a 16 bit resolution Honeywell HMC5883L magnetometer (Honeywell, Morristown, NJ, USA). With suitable printed circuit board (PCB) design, the sensor is minimized to ensure that it can be sealed in a glass tube with a diameter of 8 mm, which is shown in Figure 2. Part 2 is the coil array, including three symmetric cylindrical coils. The thickness of coils is 1 cm and the radius is 8 cm. With proper arrangement, they can suitably encircle patients' abdomens. Part 3 is the control system, containing a C8051F340 MCU and a PC. The MCU is responsible for collecting and uploading raw sensor data with a frequency of 20 Hz, as well as exciting the coils under the control of the computer. Part 4 is display unit used to display our application's interference that is shown in Figure 3 where the ultrasound scan section is demonstrated by a highlighted sector.

**Figure 4.**
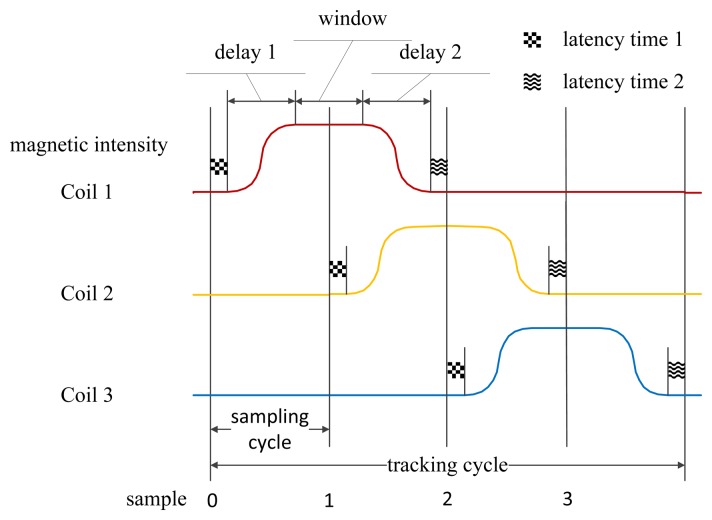
The working sequence of the ultrasonic endoscope tracking system.

A typical tracking cycle consists of four sampling cycles. An arbitrary one is demonstrated in Figure 4, in which four samples are indicated as sample 0, 1, 2 and 3. In sample 0, the sensor array outputs polluted gravitational acceleration, polluted geomagnetic flux density and polluted angular velocity. The initial estimate of orientation is gained by integrating the gyroscope's signals. Then it is converged by the outputs of the accelerator and the magnetometer, where AFQC is designed to add adaptive disposal for reducing influences of the noises. On sample *i* (*i* = 1, 2, 3), the magnetometer measures the sums of Coil *i* and geomagnetic flux density, meanwhile the orientation is continually updated by the gyroscope. By synthesizing the information of samples from 1 to 3 and the result of AFQC, we can calculate the real-time position through SEMT.

To achieve the work process above, we should strictly control the operating sequences of the three coils. Firstly, we add a latency time (latency time 1) between sample 0 and the beginning of charging Coil 1 to avoid possible disturbances. After that, a constant direct current (DC) voltage is applied on Coil 1. Because of hysteresis effects, there will be a delay (delay 1) before the magnetic intensity become constant. Then a sampling window with flat singles is available until Coil 1 begins to discharge. For control convenience, sample 1 is set at the absolute middle of sampling window. For the same reason, latency time 1 is also added between sample 1 and the beginning of charging Coil 2. Finally, after Coil 1 loses its magnetism completely and the added latency 2—which can avoid Coil 1's influences on later samples—sample 2 arrives. Because all three coils share the same electric and mechanical design, their control process has symmetry, and the sequences between sample 2 and the next tracking cycle are similar.

## 3. Orientation Algorithm

### 3.1. Nomenclature

O′ – X′Y′Z′: The body frame
O – XYZ: The earth frame
**x***^n^*: Vector in the earth frame
**x***^b^*: Vector in the body frame
**x̃**: Measured vector with noises
${\text{C}}_{m}^{n}$: Rotation matrix from frame *m* to frame *n*
**x̂**: Estimation

### 3.2. Sensor Model

*Gyroscope*: the gyroscope's output can be described as the resultant of the angular velocity **ω⃗**, the bias **b⃗** and the white noise $\vec{{\text{w}}_{\omega }}$:

(1) $$\tilde{\vec{\omega }}=\vec{\omega }+\vec{\text{b}}+\vec{{\text{w}}_{\omega }}$$

*Accelerometer*: the accelerometer's output can be described as the resultant of the gravity **g⃗** and the linear acceleration **a⃗** and the white noise $\vec{{\text{w}}_{g}}$:

(2) $$\tilde{\vec{\text{g}}}=-\vec{\text{g}}+\vec{\text{a}}+\vec{{\text{w}}_{g}}$$

*Magnetometer*: the magnetometers' output can be described as follow, where **C***_SI_* is the soft iron error matrix, and $\vec{{\text{m}}_{HI}}$ is the hard iron error, and $\vec{{\text{w}}_{m}}$ is the white noise:

(3) $$\tilde{\vec{\text{m}}}={\text{C}}_{SI}(\vec{\text{m}}+\vec{{\text{m}}_{HI}})+\vec{{\text{w}}_{m}}$$

### 3.3. Quaternion Model

Orientation is often defined as a series of rotations from the Earth frame to the body frame. Quaternion is a common method to indicate such rotations, which effectively avoid the problem of singularity in the Euler angle. In this paper, quaternion is expressed as follow:

(4) $$\text{Q}=\left[\begin{array}{c} \begin{array}{c} {\text{q}}_{0}\\ {\text{q}}_{1} \end{array}\\ \begin{array}{c} {\text{q}}_{2}\\ {\text{q}}_{3} \end{array} \end{array}\right]$$

The rotation matrix from the Earth frame to the body frame can be described in quaternion terms:

(5) $${\text{C}}_{n}^{b}\left(\text{Q}\right)=\left[\begin{array}{ccc} \begin{array}{c} {\text{q}}_{0}^{2}+{\text{q}}_{1}^{2}-{\text{q}}_{2}^{2}-{\text{q}}_{3}^{2}\\ 2\left({\text{q}}_{1}{\text{q}}_{2}+{\text{q}}_{3}{\text{q}}_{0}\right)\\ 2\left({\text{q}}_{0}{\text{q}}_{2}+{\text{q}}_{1}{\text{q}}_{3}\right) \end{array} & \begin{array}{c} 2\left({\text{q}}_{0}{\text{q}}_{3}+{\text{q}}_{1}{\text{q}}_{2}\right)\\ {\text{q}}_{0}^{2}-{\text{q}}_{1}^{2}+{\text{q}}_{2}^{2}-{\text{q}}_{3}^{2}\\ 2\left({\text{q}}_{2}{\text{q}}_{3}-{\text{q}}_{0}{\text{q}}_{1}\right) \end{array} & \begin{array}{c} 2\left({\text{q}}_{1}{\text{q}}_{3}-{\text{q}}_{0}{\text{q}}_{2}\right)\\ 2\left({\text{q}}_{0}{\text{q}}_{1}+{\text{q}}_{2}{\text{q}}_{3}\right)\\ {\text{q}}_{0}^{2}-{\text{q}}_{1}^{2}-{\text{q}}_{2}^{2}+{\text{q}}_{3}^{2} \end{array} \end{array}\right]$$

The orientation of a rigid body (e.g., the scan head of an ultrasonic endoscope) is updated through a quaternion kinetic differential Equation:

(6) $$\dot{\text{Q}}=\frac{1}{2}\text{Q}⊗\omega =\Omega \cdot \text{Q}$$

(7) $$\Omega =\frac{1}{2}\left[\begin{array}{cccc} 0 & -\omega_{x} & -\omega_{y} & -\omega_{z}\\ \omega_{x} & 0 & \omega_{z} & -\omega_{y}\\ \omega_{y} & -\omega_{z} & 0 & \omega_{x}\\ \omega_{z} & \omega_{y} & -\omega_{x} & 0 \end{array}\right]$$

where ω*_x_*, ω*_y_* and ω*_z_* are tri-axial components of the angular velocity.

According to the differential equation, the recurrence formula of quaternion in discrete sampling is deduced easily as Equation (8), in which **Ω**_t_ is comprised of the angular velocity of the moment of t, Δt is the sampling time:

(8) $${\text{Q}}_{\text{t}+\Delta \text{t}}={\text{e}}^{\Omega_{\text{t}}\cdot \Delta \text{t}}{\text{Q}}_{\text{t}}$$

### 3.4. AFQC Structure

In the AFQC algorithm, the estimations—updated by the gyroscope—are converged by the accelerometer and magnetometer signals. Self-adaptive weight factors are designed to judge discrepancies between sensor signals and true values of the gravity and geomagnetic field.

We introduce an estimation matrix $\hat{{\text{C}}_{n}^{b}}$, which is structured by quaternion as Equation (5). Traditionally, to observe the estimation error, the minimum-squared-error criterion is used. The error function E is defined as [25]:

(9) $$\text{F}\;=\left[\begin{array}{c} \tilde{\vec{{\text{g}}^{b}}}-\hat{{\text{C}}_{n}^{b}}\vec{{\text{g}}^{n}}\\ \tilde{\vec{{\text{m}}^{b}}}-\hat{{\text{C}}_{n}^{b}}\vec{{\text{m}}^{n}} \end{array}\right]$$

(10) $$\text{E}\;={\text{F}}^{\text{T}}\cdot \text{F}$$

where:

$\tilde{\vec{{\text{g}}^{b}}}$: is a 3 × 1 vector of the accelerometer output in the body frame.
$\tilde{\vec{{\text{m}}^{b}}}$: is a 3 × 1 vector of the magnetometer output in the body frame.
$\vec{{\text{g}}^{n}}$: is a 3 × 1 vector of the true gravity value in the Earth frame.
$\vec{{\text{m}}^{n}}$: is a 3 × 1 vector of the true geomagnetic value in the Earth frame.

By minimizing the error, the orientation estimation will be converted. However, according to the sensor model above, the accelerometer and the magnetometer don't strictly output the true gravity value and the true geomagnetic value, which will decrease the accuracy or even invalidate the convergence. To solve such problems, we build an adaptive error function **E***_ad_*;

(11) $${\text{E}}_{ad}=\left\|\frac{\left\|\tilde{\vec{{\text{m}}^{b}}}\right\|-\left\|\vec{{\text{m}}^{n}}\right\|}{\left\|\vec{{\text{m}}^{n}}\right\|}\right\|{\left(\tilde{\vec{{\text{g}}^{b}}}-\hat{{\text{C}}_{n}^{b}}\vec{{\text{g}}^{n}}\right)}^{2}+\left\|\frac{\left\|\tilde{\vec{{\text{g}}^{b}}}\right\|-\left\|\vec{{\text{g}}^{n}}\right\|}{\left\|\vec{{\text{g}}^{n}}\right\|}\right\|{\left(\tilde{\vec{{\text{m}}^{b}}}-\hat{{\text{C}}_{n}^{b}}\vec{{\text{m}}^{n}}\right)}^{2}$$

In the corrected error function, $\left\|\frac{\left\|\tilde{\vec{{\text{m}}^{b}}}\right\|-\left\|\vec{{\text{m}}^{n}}\right\|}{\left\|\vec{{\text{m}}^{n}}\right\|}\right\|$ distinguishes the difference between magnetometer signals and true geomagnetic values by comparing their relative magnitudes, which functions as the judgment of the magnetometer's reliability in AFQC. When magnetic disturbances exist, $\left\|\frac{\left\|\tilde{\vec{{\text{m}}^{b}}}\right\|-\left\|\vec{{\text{m}}^{n}}\right\|}{\left\|\vec{{\text{m}}^{n}}\right\|}\right\|$ increases the weight of ${\left(\tilde{\vec{{\text{g}}^{b}}}-\hat{{\text{C}}_{n}^{b}}\vec{{\text{g}}^{n}}\right)}^{2}$. As a result, the accelerometer signals will be dominant in the convergence process, and the role of the magnetometer output will be weakened. Similarly, when the endoscope is accelerating, the outputs of the magnetometer will dominate the convergence, which can effectively reduce the impact of linear acceleration to attitude algorithm.

On the basis of the adaptive error function, a new objective function can be easily built as:

(12) $${\text{F}}_{ab}=\left[\begin{array}{c} \sqrt{\left\|\frac{\left\|\tilde{\vec{{\text{m}}^{\text{b}}}}\right\|-\left\|\vec{{\text{m}}^{\text{n}}}\right\|}{\left\|\vec{{\text{m}}^{\text{n}}}\right\|}\right\|}\left(\tilde{\vec{{\text{g}}^{\text{b}}}}-\hat{{\text{C}}_{\text{n}}^{\text{b}}}\vec{{\text{g}}^{\text{n}}}\right)\\ \sqrt{\left\|\frac{\left\|\tilde{\vec{{\text{g}}^{\text{b}}}}\right\|-\left\|\vec{{\text{g}}^{\text{n}}}\right\|}{\left\|\vec{{\text{g}}^{\text{n}}}\right\|}\right\|}\left(\tilde{\vec{{\text{m}}^{\text{b}}}}-\hat{{\text{C}}_{\text{n}}^{\text{b}}}\vec{{\text{m}}^{\text{n}}}\right) \end{array}\right]$$

G-N method can minimize the error conveniently, which is expressed as:

(13) $${\hat{\text{Q}}}_{k}^{n+1}={\hat{\text{Q}}}_{k}^{n}-\alpha \langle {\left[{\text{J}}_{ad}^{\text{T}}({\hat{\text{Q}}}_{k}^{n})J_{ad}({\hat{\text{Q}}}_{k}^{n})\right]}^{-1}{\text{J}}_{ad}^{\text{T}}({\hat{\text{Q}}}_{k}^{n}){\text{F}}_{\text{ab}}({\hat{\text{Q}}}_{k}^{n})\rangle$$

(14) $${\text{J}}_{ad}({\hat{\text{Q}}}_{k}^{n})=\frac{d{\text{F}}_{ab}({\hat{\text{Q}}}_{k}^{n})}{d{\hat{\text{Q}}}_{k}^{n}}$$

where ${\hat{\text{Q}}}_{k}^{n}$ is the result of the *n*th G-N convergence in the *k*th tracking cycle.

Because nonlinear optimizations are very sensitive to initial values, if the selection is undeserved, only local minima or diverged results will be obtained. In the *k*th tracking cycle, after convergence, the estimation of the orientation is $\hat{{\text{Q}}_{k}}$. At the moment of *k*·4Δt + *i*Δt(*i* = 0, 1, 2, 3), the **Ω** matrices constructed by the corresponding angular velocities are **Ω***_k_*_·4Δt+_*_i_*_Δt_. According to Equation (8), the initial value ${\text{Q}}_{k+1}^{\text{ini}}$ of the *k*+1th tracking cycle follows as:

(15) $${\text{Q}}_{k+1}^{\text{ini}}={\text{e}}^{\sum_{i=0}^{3}\Omega_{(k+i)\cdot \Delta \text{t}}\cdot \Delta \text{t}}\hat{{\text{Q}}_{k}}$$

In a short period of time, the cumulative error of gyroscope caused by the bias is not obvious, so that the initial value of the *k* + 1th cycle is close enough to the true value, which can ensure the effectiveness of the convergence.

## 4. Position Algorithm

### 4.1. Modeling of Circular Coil

**Figure 5.**
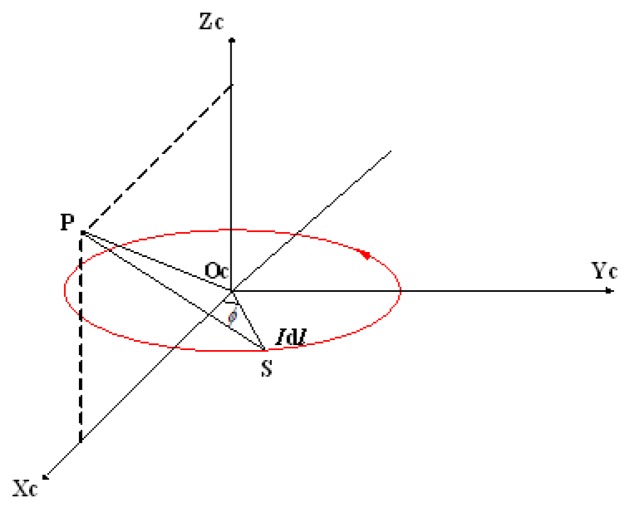
Analyzing on the magnetic field of a single turn circular coil.

For a single turn circular coil—whose radius is *a* and carrying current is I —and an arbitrary point *P*, the orthogonal coordinate system O_C_ − X_C_Y_C_Z_C_ is built, which is shown in Figure 5. The base vectors of three axes are $\vec{{\text{e}}_{x}}$, $\vec{{\text{e}}_{y}}$, and $\vec{{\text{e}}_{z}}$. O_C_ is located at the center of the coil. X_C_ axis and Y_C_ axis are along radial directions of the coil. Z_C_ axis is perpendicular to the coil. *P* is at the plane X*_C_*Y*_C_*Z*_C_* and its coordinate is (x, 0, z).

We choose a point *S* on the coil, whose coordinates are (acosϕ, asinϕ, 0). Considering that the permeability of the human body is very close to that of air, the magnetic field of the current element Id**l⃗** which located at *S* can be calculated by the Biot-Savart law as follows:

(16) $$\text{d}\vec{\text{B}}=\frac{\mu_{0}}{4\pi }\frac{\text{Id}\vec{\text{l}}\times \vec{\text{r}}}{{\text{r}}^{3}}$$

(17) $$\vec{\text{r}}=\left(\text{x}-\text{acos}\phi \right)\cdot \vec{{\text{e}}_{x}}-\text{asin}\phi \cdot \vec{{\text{e}}_{y}}+\text{z}\cdot \vec{{\text{e}}_{z}}$$

(18) $$\text{d}\vec{\text{l}}=a(-\text{sin}\phi \cdot \vec{{\text{e}}_{x}}+\text{cos}\phi \cdot \vec{{\text{e}}_{y}})\text{d}\phi$$

where **r⃗** is the vector from *S* to *P*, and *μ*_0_ is the permeability of air.

Integrating along the closed loop, the magnetic flux density at *P* exited by the coil is as follows:

(19) $$\vec{\text{B}}=\frac{\mu_{0}\text{Ia}}{4\pi }\int_{0}^{2\pi }\frac{\text{zcos}\phi \cdot \vec{{\text{e}}_{\text{x}}}+\text{zsin}\phi \cdot \vec{{\text{e}}_{\text{y}}}+\left(\text{a}-\text{xcos}\phi \right)\cdot \vec{{\text{e}}_{\text{z}}}}{{\left({\text{z}}^{2}+{\text{a}}^{2}+{\text{x}}^{2}-2\text{axcos}\phi \right)}^{\frac{3}{2}}}\text{d}\phi$$

In which the component along the *Y_C_* axis can be expressed by:

(20) $${\vec{\text{B}}}_{\text{y}}=\frac{\mu_{0}\text{Ia}}{4\pi }\int_{0}^{2\pi }\frac{\text{zsin}\phi }{{\left({\text{z}}^{2}+{\text{a}}^{2}+{\text{x}}^{2}-2\text{axcos}\phi \right)}^{\frac{3}{2}}}\text{d}\phi =0$$

From above, we can conclude that for any point in space, the magnetic flux density excited by a single turn circular coil is parallel to one specific plane, which is through the selected point and the center of the coil, and perpendicular to the coil. We define this plane as the target plane.

A symmetric cylindrical coil consists of many single turned circular coils. These single turn coils are parallel to each other and share one midperpendicular. Therefore target planes of the single turn circular coils strictly overlap, and any of these planes can be defined as the target plane of the cylindrical coil.

### 4.2. SEMT Structure

We apply the general conclusions above to our localization system. In the Earth frame, the coordinates of the geometric centers of three coils are (x*_i_*, y*_i_*, z*_i_*) (*i* = 1,2,3), and the cross sections through the coils' centers are as follows:

(21) $${\text{A}}_{i}\left(\text{x}-{\text{x}}_{i}\right)+{\text{B}}_{i}\left(\text{y}-{\text{y}}_{i}\right)+{\text{C}}_{i}\left(\text{z}-{\text{z}}_{i}\right)=0\left(i=1,2,3\right)$$

where (x, y, z) is an arbitrary point on the cross sections, and (A*_i_*, B*_i_*, C*_i_*) is one of the cross sections' normal vectors.

In the *k*th positioning cycle, after convergence, the estimation of orientation through AFQC is $\hat{{\text{Q}}_{k}}$. At the moment of *k*·4Δt + *i*Δt, **Ω** Matrix obtained by the gyroscope is **Ω***_k_*_·4Δt +_ *_i_*_Δt_ respectively, and the rotation matrix from the body frame to the Earth frame is expressed as follows according to Equation (8):

(22) $${\text{C}}_{n_{k\cdot 4\Delta t+i\Delta t}}^{b}={\text{e}}^{\sum_{j=0}^{i-1}\Omega_{k\cdot j\Delta \text{t}+\Delta \text{t}}\cdot \Delta \text{t}}\hat{{\text{Q}}_{k}}\left(i=1,2,3\right)$$

In the body frame, the output of the magnetometer at the *k*·4Δt + *i*Δt moment is $\vec{{\text{m}}_{C+G_{k\cdot 4\Delta t+i\Delta t}}^{b}}$, which is the sum of magnetic flux density of Coil *i* and the Earth in the body frame. Transforming it to the Earth frame we can calculate Coil *i*'s magnetic flux density by Equation (23):

(23) $$\vec{{\text{m}}_{C_{k\cdot 4\Delta t+i\Delta t}}^{n}}={\left({\text{C}}_{n_{k\cdot 4\Delta t+i\Delta t}}^{b}\right)}^{\text{T}}\cdot \vec{{\text{m}}_{C+G_{k\cdot 4\Delta t+i\Delta t}}^{b}}-\vec{{\text{m}}^{n}}\left(i=1,2,3\right)$$

According to the definition of target planes, the normal vector of Coil *i*'s target plane at the *k*·4Δt + *i*Δt moment is both perpendicular to its magnetic flux density and normal vector, so the normal vector of this plane can be calculated as follows:

(24) $$\left(\begin{array}{ccc} {\text{A}}_{k\cdot 4\Delta t+i\Delta t}^{*}, & {\text{B}}_{k\cdot 4\Delta t+i\Delta t}^{*}, & {\text{C}}_{k\cdot 4\Delta t+i\Delta t}^{*} \end{array}\right)=\left(\begin{array}{ccc} {\text{A}}_{i}, & {\text{B}}_{i}, & {\text{C}}_{i} \end{array}\right)\times {\vec{{\text{m}}_{C}^{n}}}_{k\cdot 4\Delta t+i\Delta t}\left(\text{i}=1,2,3\right)$$

Considering the target plane is through the center of Coil *i*, the target plane of Coil *i* is:

(25) $${\text{A}}_{k\cdot 4\Delta t+i\Delta t}^{*}\left(\text{x}-{\text{x}}_{i}\right)+{\text{B}}_{k\cdot 4\Delta t+i\Delta t}^{*}\left(\text{y}-{\text{y}}_{i}\right)+{\text{C}}_{k\cdot 4\Delta t+i\Delta t}^{*}\left(\text{z}-{\text{z}}_{i}\right)=0\left(i=1,2,3\right)$$

The three target planes of three coils intersect at the point where the sensor located. Therefore the position of the sensor can be calculated by solving the simultaneous equations of the three target planes, expressed by Equation (25).

### 4.3. Accuracy Analysis for SEMT

**Figure 6.**
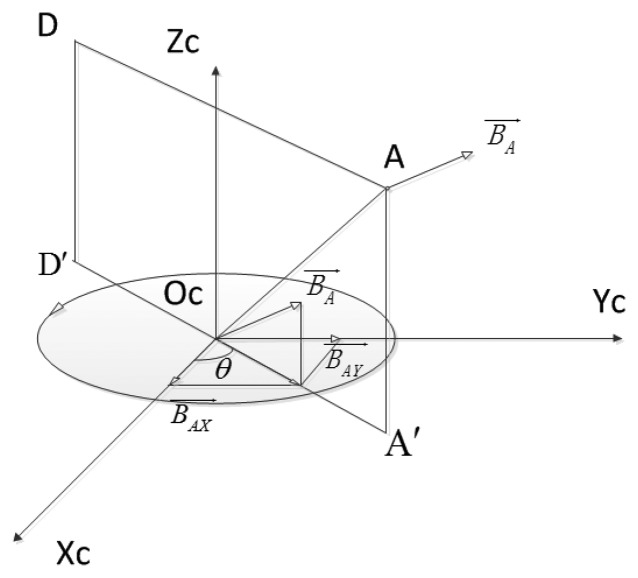
Analyzing on errors of SEMT.

In real working space, sensors' inherent noises and magnetic distortions are unavoidable. These factors can disturb the determinations of target planes, and consequently decrease SEMT's accuracy. To guarantee good performance during tracking, SEMT's response to noises and distortions should be analyzed carefully. We still adopt the model of Figure 5 for illustrating SEMT's stability, where another point *A* is chosen, as shown in Figure 6.

The magnetic flux density on A excited by the coil is $\vec{{\text{B}}_{\text{A}}}$, which can be expressed as (B_Ax_, B_Ay_, B_Az_). The target plane of *A* is AA′D′D. If the angle formed by $\vec{{\text{B}}_{\text{A}}}$'s horizontal component and X*_C_* axis—defined as θ—is determined, the target plane is also solely determined. This angle θ is expressed as:

(26) $$\theta =\text{actan}\frac{{\text{B}}_{\text{Ay}}}{{\text{B}}_{\text{Ax}}}$$

Exerting a small disturbance ΔB*_Ax_* to B*_Ax_*, θ changes to θ + Δθ accordingly. Δθ/ΔB*_Ax_* can be introduced to determine the disturbing degree of B*_Ax_* to θ, which then determine the disturbing degree of B_Ax_ to the target plane. If $\left\|\frac{\partial \theta }{\partial {\text{B}}_{Ax}}\right\|$ is larger, disturb of B*_Ax_* to the target plane is more evident. Similarly, $\left\|\frac{\partial \theta }{\partial {\text{B}}_{Ay}}\right\|$ can indicate the disturbing degree of B*_Ay_* to the target plane:

(27) $$\left\|\frac{\partial \theta }{\partial {\text{B}}_{Ax}}\right\|={\left({\text{B}}_{Ay}+\frac{{{\text{B}}_{Ax}}^{2}}{{\text{B}}_{Ay}}\right)}^{-1}$$

(28) $$\left\|\frac{\partial \theta }{\partial {\text{B}}_{Ay}}\right\|={\left({\text{B}}_{Ax}+\frac{{{\text{B}}_{Ay}}^{2}}{{\text{B}}_{Ax}}\right)}^{-1}$$

From Equations (27) and (28) we reach that when B*_Ax_* or B*_Ay_* is smaller; the degree of their disturbance of the determination of the target plane is larger. When the sensor is near the axis wire of the coil, B*_Ax_* and B*_Ay_* are both very small, the noises of the magnetometer will affect the determination of θ in the greatest extent, which in turn disturbs the output of the target plane, and eventually increases the positioning errors. Namely, the area near the coils' axes has the biggest error for position determination. As a result, we should exclude the axis wires of the coils in tracking area.

In addition, if the three target planes meet at a straight line, the algorithm fails. Such a condition will form singular points for localization. Hence in the design of the coils' layout style, we should ensure that the singular points are also out of the tracking area.

Apart from the coils' location, the sampling cycle can influence SEMT's accuracy too. Theoretically speaking, in the process of motivating three coils, the position of the sensor must be basically unchanged, and the gyroscope's orientation estimates should be adequately accurate. For an ultrasonic endoscope, which moves in the GI tract with low speed, as long as the sampling time Δt is small enough, the conditions are easy to implement. The confine of the sampling time is in conformity with the attitude tracking requirements.

To sum up, two elements are essential for the SEMT's accuracy. The first is a reasonable layout style for the three coils, which means the axis wires and singular points should be out of the position area. The second is a short enough sampling cycle which is particularly desirable in dynamic tracking.

### 4.4. Integrated 6-DOF Tracking

**Figure 7.**
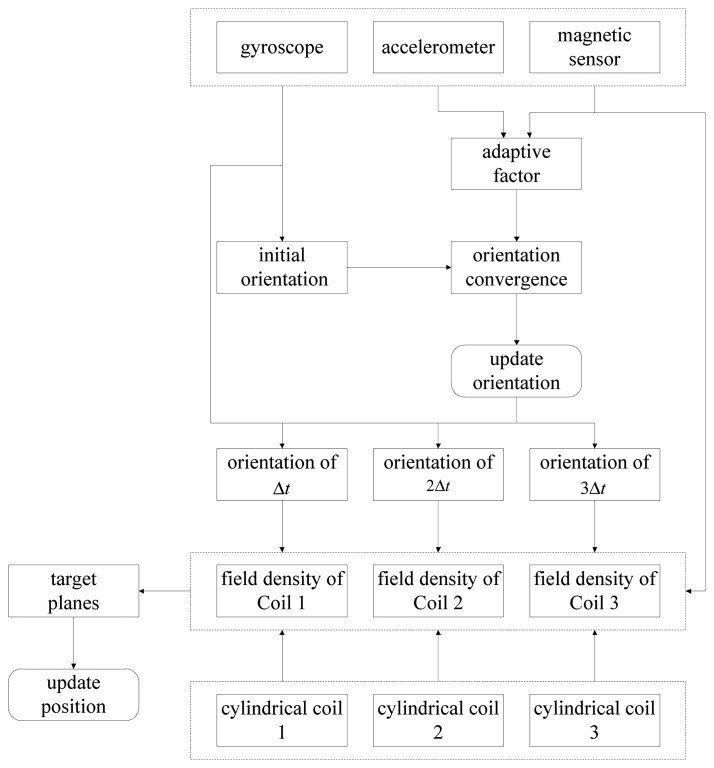
Tracking solution for ultrasonic endoscopes.

From the above, a complete 6-DOF positioning scheme is concluded as illustrated in Figure 7. Through additional adaptive processing, G-N converge will achieve good anti-noise characters; and by synthesizing orientation estimates by INS and EMT technology, the positioning method is greatly simplified.

## 5. Experimental Methods

### 5.1. Experimental Equipment

**Figure 8.**
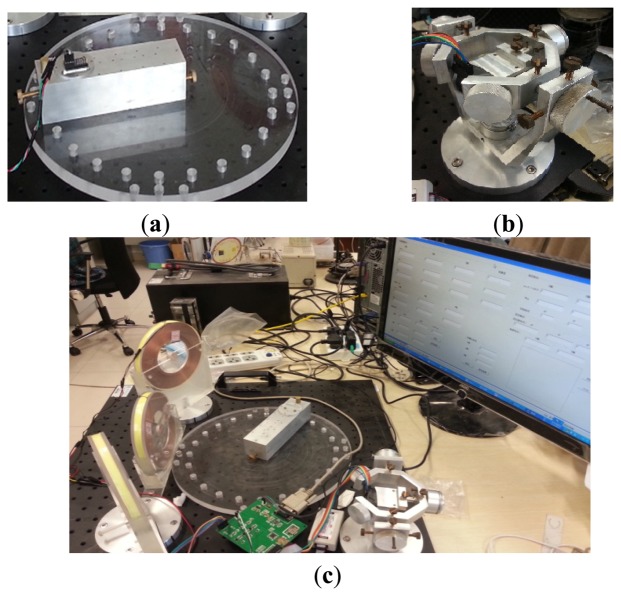
(**a**) The assessment device; (**b**) The three-axis turntable; (**c**) The experimental platform.

We designed an assessment device by simulating Hummel's scheme [31] to evaluate our localization results. The device can provide standardized attitude angle values every 15° from 0° to 360° and standardized position values on concentric circles with radii of 2.5, 5, 7.5, and 10 cm every 15° The experimental positioning range was bigger than the abdominal cavity volume of adults. We also introduced a three-axis turntable to assess orientation estimation in dynamic state. Axes of the turntable were equated with potentiometers. The standard values of orientation can be obtained by A/D conversion (Figure 8).

### 5.2. Experimental Procedure

The complete experiment consisted of four parts. In the first experiment, we recorded root-mean-square error (RMSE) of AFQC with Euler angle in static and dynamic states, where a classical G-N method was added as a comparison group [25].

In the second experiment, the anti-interference ability of AFQC was tested in static tracking. Firstly a common laparoscopic instrument was put close to the sensor during tracking to apply magnetic interference, and its magnitude was recorded through the magnetometer's signals. Then the sensor was pushed to exert accelerated interference, whose magnitude was recorded by the accelerometer's signals. The static RMSE under interferences were acquired to quantify performances of AFQC. G-N convergence without adaptive complement acted as a comparison group too [25].

In the third experiment, the positioning accuracy of SEMT was tested. We designed two kinds of coil layout styles to verify the relationship between noise responses of SEMT and the coils' display methods. For both styles, the outputs of SEMT were sampled 20 times at each point to obtain the RMSE.

**Figure 9.**
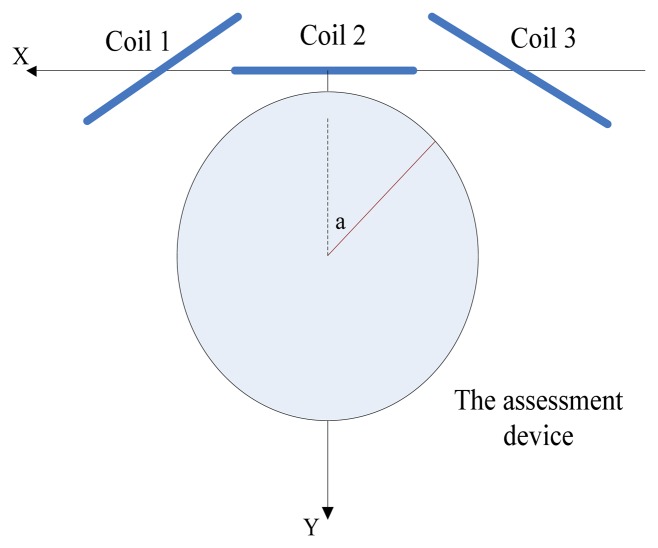
The first layout style of the experimental equipment (top view).

The first style was demonstrated as Figure 9. Thee coils were all perpendicular to the horizontal plane, and the Earth frame's origin was located at the geometric center of Coil2. From Coil 1 to Coil 3, their cross sections were expressed as Equations (29)–(31), respectively, which were all obtained in centimeters:

(29) $$-\frac{1}{2}\left(\text{x}-17.5\right)+\frac{\sqrt{3}}{2}\left(\text{y}-8\right)=0$$

(30) y=0

(31) $$\frac{1}{2}\left(\text{x}+17.5\right)+\frac{\sqrt{3}}{2}\left(\text{y}-5\right)=0$$

Accordingly, obverted points' coordinates on the assessment device were Equations (32) to (34):

(32) x=−r⋅sin(j⋅π/6)

(33) y=17.5−r⋅cos(j⋅π/6)

(34) z=−1.5(r=2.5,5,7.5,10;j=0,1,…11)

**Figure 10.**
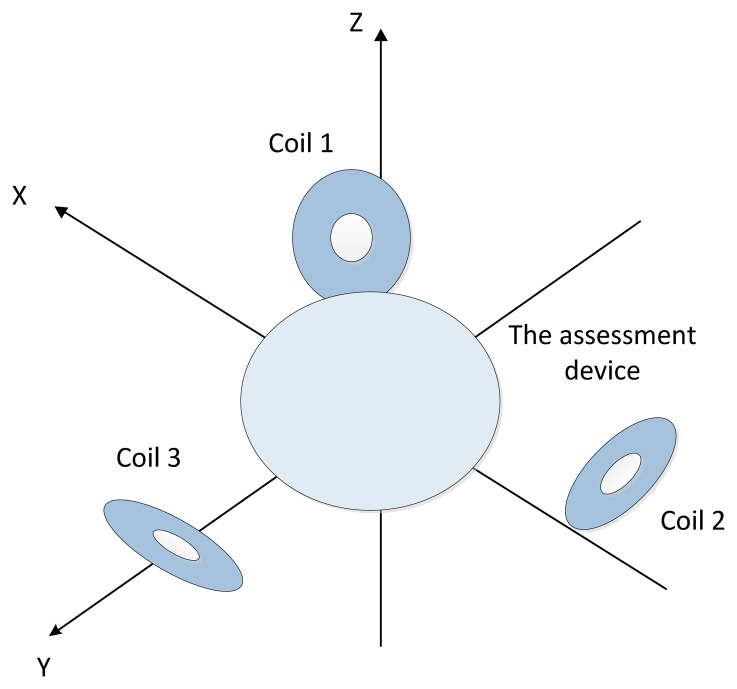
The second layout style of the experimental equipment.

The second style was as demonstrated in Figure 10. Three coils were expressed as Equations (35)–(37), and measured points were Equations (38)–(40). All coordinates were obtained in centimeters too:

(35) $$-\frac{\sqrt{3}}{4}\left(x-\frac{25}{2}\sqrt{3}\right)+\frac{1}{4}\left(y+\frac{25}{2}\right)+\frac{\sqrt{3}}{2}\left(z-\frac{12+\sqrt{3}}{3}\right)=0$$

(36) $$\frac{\sqrt{3}}{4}\left(x+\frac{25}{2}\sqrt{3}\right)+\frac{1}{4}\left(y+\frac{25}{2}\right)+\frac{\sqrt{3}}{2}\left(z-\frac{12+\sqrt{3}}{3}\right)=0$$

(37) $$-\frac{1}{2}\left(y-25\right)+\frac{\sqrt{3}}{2}\left(z-\frac{12+\sqrt{3}}{3}\right)=0$$

(38) x=r⋅sin(j⋅π/6)

(39) y=r⋅cos(j⋅π/6)

(40) z=7(r=2.5,5,7.5,10;j=0,1,…11)

In the fourth experiment, influences of the sampling cycle on the accuracy in dynamic tracking were tested. In the algorithm structure sections we have proven that the sampling cycle can affect the accuracy of both AFQC and SEMT. To measure specific relations between them, a group of contrast experiments were carried out, in which sampling cycles were set as 0.05, 0.06, 0.07…0.14 s. For every different cycle, results of roll angle and positional measurement on *X* axis were recorded, and the corresponding absolute errors were then worked out.

## 6. Results and Discussion

### 6.1. Orientation Measurement

Table 1 demonstrates the RMSE of AFQC and traditional G-N algorithm on orientation estimation without disturbances. As can be seen from the table, the RMSE in static state of the two algorithms are essentially equal on three Euler angles. However in dynamic state, the performances of AFQC are better than G-N, which is caused by the inhibitory effect of AFQC on accelerations in the convergence process.

**Figure 11.**
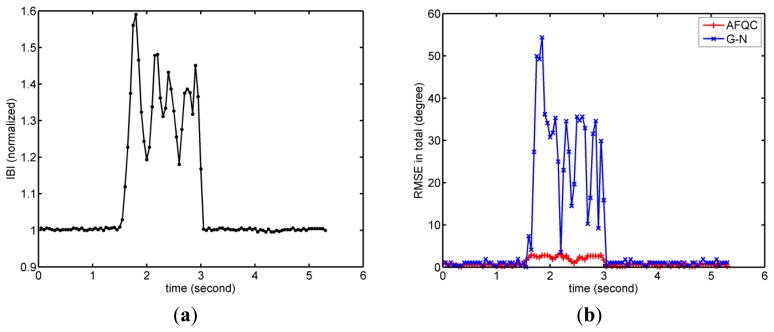
Typical results for estimation errors when magnetic disturbances applied. (**a**) Magnetic field magnitude. Magnetometer's signals become variable when magnetic field is disturbed; (**b**) RMSE of AFQC and G-N.

**Figure 12.**
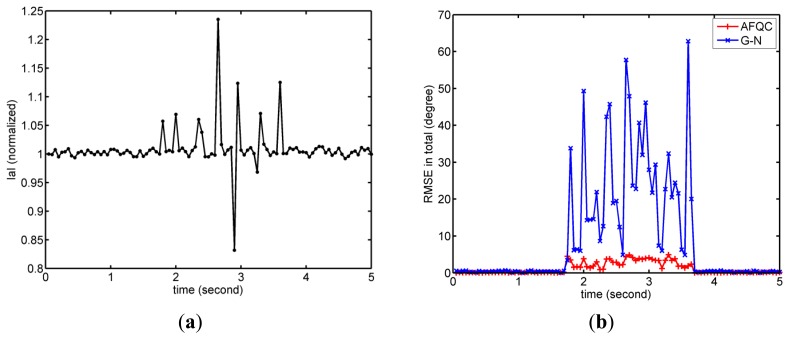
Typical results for estimation errors when acceleration disturbances applied. (**a**) Magnitude of specific force. Acceleration's signals become variable when the sensor is moving; (**b**) Orientation outputs of AFQC and G-N.

In the second experiment, noises' magnitudes and relevant RMSE of AFQC and G-N were shown in Figures 11 and 12. No matter the acceleration or magnetic disturbances exerted, AFQC can basically control the noises and obtain stable outputs, while the non-adaptive algorithm fails. Specific RMSE of AFQC in the present of disturbances is recorded in Table 2. In such circumstances, total RMSE of AFQC is controlled under 3.5°

Our orientation estimation algorithm is verified in a wide variety of environments (static, dynamic, with or without disturbances). Without interferences, AFQC can output stable and effective results. Although acceleration or magnetic interferences exist, errors can be controlled to ensure accurate results.

It is worth noting that in INS many algorithms are built on the convergence through double vectors without adaptive characteristics [25–27], which will eventually fail when applying noises. At the same time, the uncertainty of magnetic noises also limits anti-interference algorithms attempting to build Markov models of sensor output pattern [29]. AFQC avoids pattern analysis which involves complex theoretical assumptions, and still ensures enough anti-jamming capability. Compared with EMT tracking methods reported by other groups [8–10], we have achieved higher accuracy in a clean environment. Disturbances are also considered in our method, which are ignored in the papers above. When disturbances exist, an accuracy of 3.5° is much higher than NDI's 10° reported by Ren [11].

Essentially, AFQC judges the reliability of the acceleration and magnetometer by comparing the magnitudes of their outputs with theoretical values. However, gravitational acceleration and magnetic flux density are vectors; magnitudes cannot strictly characterize disturbances. Considering our experimental results, we still can safely conclude the adaptive processing is enough for endoscopic tracking.

### 6.2. Position Measurement

**Figure 13.**
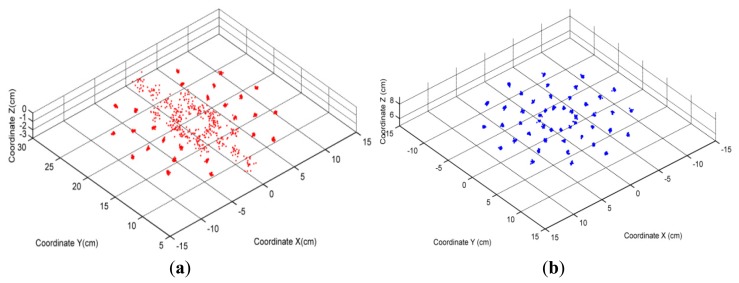
The results of the positioning experiment. (**a**) Results from the first layout style; (**b**) Results from the second layout style.

The tracking results for both two layout styles are shown in Figure 13. We can find the great differences between them. For the first layout style, distribution of SEMT's results is bimodal. On twenty-six points—which are defined as group 1—measurements for the same spot are less dispersive between each other. Meanwhile on other twenty-two points (group 2), dispersions are much larger. For the second layout style, all forty-eight points share similar distributions which are almost the same as group 1 in the first style.

**Figure 14.**
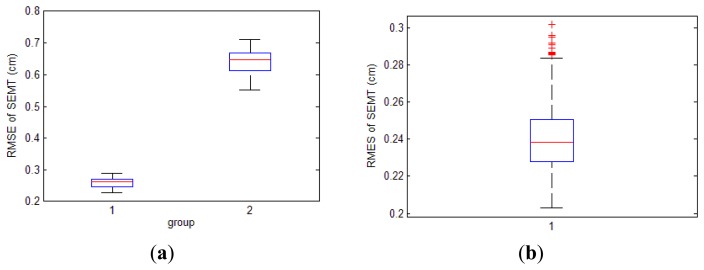
Distribution of SEMT's errors. (**a**) Errors of the first layout style. Group 1 consists of the twenty-six points of higher accuracy, and group 2 consists of the other twenty two with lower accuracy; (**b**) Errors of the second layout style.

To reveal their differences mathematically, we carry out RMSE on every point for both layout styles, and demonstrate them by the box plots shown in Figure 14. For the first style, we can find that group 1 is not only less dispersive than group 2, but also more focused on the real values. Experimental results from the second style are all well focused on the true values, making measurements much more stable and trustworthy.

Apart from the box plot, specific average values and standard deviations for SEMT's errors in these two styles are also recorded in Tables 3 and 4. Summarizing from the two tables, the first layout style's error is about 0.7 cm, while the second style's is within 0.3 cm.

It is the relative position between the observed area and the axes of the coils that cause the differences of the two styles. When adopting the first style, coils' axes traverse the tracking area; and measurements around axes are badly influenced as we have already proved. These points constitute group 2, whose errors are up to 7 cm. Meanwhile, other points away from the axes still offer satisfactory precision, and they are classified into group 1. However, by setting the layout method more wisely, the axes of three coils can be absolutely excluded from our position region as the second style does. Although the position area is unchanged, the second style can improve tracking results markedly and be directly adopted for practical application.

Compared with accuracy reported by other groups [32,33], the accuracy of 0.3 cm is a bit lower, but it is still small and within acceptable limits [34,35]. On the other hand, in contrast with their large array which contains dozens of coils and specialized high-speed circuits, our system structure is much more simplified, and only requires a low-cost integrated sensor, regular coils and basic hardware. Besides, SEMT contains no complex iterations which are commonly applied by them, so bulk calculating costs are effectively avoided. In a word, SEMT can achieve an effective accuracy through much lower systematic complexity in position estimation.

### 6.3. Influences of Sampling Cycle on Accuracy

**Figure 15.**
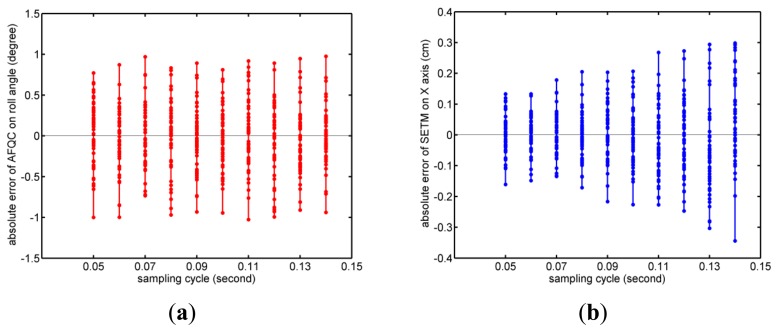
Absolute error of tracking for different sampling cycles. (**a**) RMSE of AFQC on roll angle; (**b**) RMSE of SEMT on *X* axis.

Results of the fourth experiment are demonstrated in Figure 15. From them, we find that the accuracy of orientation estimate remains almost unchanged when the sampling cycles become longer. Meanwhile, the accuracy of position determination decreases, and it is even more obvious when the cycle is longer than 0.11 s.

For AFQC, orientation estimates based on the gyroscope only provide an initial value, which is then converged by the accelerometer and the magnetometer's signals. As long as the gyroscope's accumulative errors are small enough to guarantee the error function's local minimum—obtained by convergences—is the global minimum, errors caused by the gyroscope can be effectively controlled. From the experimental results, we find this condition is easy to achieve.

However, SEMT's situation is much more different. When obtaining analytic forms of target planes in the Earth frame, real-time orientation is needed. Because of the external fields generated by the coils, we cannot obtain the orientation from double-vector convergences. Outputs of the gyroscope are the only sources for estimation, so its bias will restrict the accuracy. What's more, sensors' movement between coils' excitation can change the magnetometer's measuring point, which will impact resolving target planes, and finally damage SEMT. Therefore, when the length of sampling cycle is beyond a certain value (in our system, it is 0.11 s), it is sufficient to disturb determination of target planes and lower SEMT's overall accuracy. Despite the advantages of improving the accuracy, it is impractical to reduce the sampling cycle further. This is because data sampling and coils' control are highly coordinated as motioned in the System Architecture section, and the inherent hysteresis in the materials used limits the minimum value of the sampling cycle. As a result, a sampling cycle of 0.05 s is the optimal choice for our system.

### 6.4. Contributions Relative to Similar Research

Similar works are mainly reported by Ren in [11,19,20]. In his research, Ren combines a custom EMT subsystem designed in [32,33]—which contains a generator array bearing 48 coils and a receiving array bearing three coils—with a typical MARG sensor. Normally, the MARG provides the orientation estimates. If the acceleration or magnetic distortion are too high, the EMT subsystem will replace MARG to determine orientation. Then, in combination with the orientation result, MARG derives the real-time position through the kinematic relationship between position, velocity and acceleration. Compared with his work, we believe we have achieved at least three improvements.

Firstly, we propose a new orientation estimation algorithm with anti-interference ability to resist acceleration and magnetic distortion. In Ren's solution, as EMT subsystem is adopted to reduce the influences from disturbances, and it needs to be very robust. To achieve this requirement, its hardware structure is very complex, including a huge generator array. The estimation algorithm, which includes complex strategies like Bayesian filtering [32] or particle filtering [33], is also very costly. On the contrary, our solution is much easier to accomplish. Only a low-cost MARG sensor and a solution for a basic least squares problem are required. Although the processes in hardware and algorithm are both largely predigested, the accuracy we get is impressive, especially with interferences.

Secondly, we design a novel fusion method for position tracking and handle its potential shortages in a more detailed manner. In Ren's papers, position results are essentially determined through the double integral of the accelerator's output. In theory, inherent noises in the accelerator would decrease the sensor's signal-to-noise ratio, and devaluate the positional measurements. Unfortunately, Ren neither analyzes this potential threat nor provides an effective method to avoid it. On the contrary, when integrating magnetic measurements with orientation obtained by MARG, we put forward some factors that might influence its accuracy; it includes layout styles for coils and sampling cycle lengths. Then we evaluate and verify these inferences through sets of experiments. Based on them, optimal choices are proposed. As a result, errors are further controlled.

Thirdly, we greatly simplify the EMT-INS hybrid system which can solve the contradiction between a tracking system's complexity and clinical practicability [16]. In an orientation estimate, an easy Gauss-Newton method with adaptive weights is proved sufficient for reliable outputs with anti-interference ability. In position determination, a generator array with only three coils is needed, and the corresponding fusion algorithm only including three linear equations is also very convenient for resolving.

### 6.5. Deficiencies and Future Work

There remain some limits to our system. First of all, accuracy for positional tracking is relatively low. We suppose this is caused by the coil manufacturing technique. During tracking, only if every coil is cylindrically symmetrical around every radial direction, can the determination for a target plane be totally accurate. It is a condition that only can be approached but not reached, so the accuracy we obtain is bounded by it. Second, magnetic distortion is still not overcome completely, especially in positional tracking. Currently, we just optimize SEMT's stability through avoiding points which are vulnerable to magnetic disturbances, so more active methods that can resist magnetic distortion are required in the process for better performances.

Aiming at these deficiencies, we will firstly improve our process of manufacturing the generators in order to achieve higher accuracy for SEMT. At the same time, active anti-interference techniques should be researched intensively, where more advanced hardware and algorithm research would be both desirable.

## 7. Conclusions

In summary, this paper develops a novel EMT-INS hybrid tracking system for ultrasonic endoscopes, where integrated algorithm and hardware prototype are both covered. For orientation estimates, a low-cost MARG is introduced. By adding two adaptive weights that can adjust themselves automatically according to acceleration and magnetic distortion, we successfully provide the traditional Gauss-Newton method with new anti-inference ability.

For position determination, EMT subsystems are highly simplified. An easy hardware design with only three coils has been achieved. Accordingly, a new hybrid strategy named SEM can effectively fuse sensing information from the two subsystems. To obtain more stable output, factors relative to SEMT's accuracy are also carefully studied. On the whole, by fusing two sensing systems, we partly solve the instability within traditional EMT system, and also reduce its complexity in both the hardware and algorithm aspects.

## Figures and Tables

**Table 1.** The RMSE of AFQC and N-G without disturbances.

**Euler Parameter**	**AFQC**	**G-N**
RMSE (pitch angle) static	0.694°	0.676°
RMSE (pitch angle) dynamic	0.753°	0.823°
RMSE (roll angle) static	0.696°	0.701°
RMSE (roll angle) dynamic	0.723°	0.814°
RMSE (heading angle) static	1.069°	1.127°
RMSE (heading angle) dynamic	1.168°	1.563°

**Table 2.** The RMSE of AFQC with disturbances.

**Euler Parameter**	**Magnetic Disturbances**	**Acceleration Disturbances**
RMSE (pitch angle)	1.2205°	1.5948°
RMSE (roll angle)	1.5608°	1.5551°
RMSE (heading angle)	1.6877°	2.1288°
RMSE (in total)	2.6027°	3.0764°

**Table 3.** The error distribution of SEMT in the first layout style.

**RMSE**	**Group 1**	**Group 2**
average value	0.2599 cm	0.6365 cm
standard deviation	0.0170 cm	0.0433 cm

**Table 4.** The error distribution of SEMT in the second layout style.

**RMSE**	**Value**
average value	0.2396 cm
standard deviation	0.0166 cm
